# Role of plasma angiogenesis factors in the efficacy of first‐line chemotherapy combined with biologics in *RAS* wild‐type metastatic colorectal cancer: Results from the GI‐SCREEN CRC‐Ukit study

**DOI:** 10.1002/cam4.6486

**Published:** 2023-08-28

**Authors:** Satoshi Yuki, Kentaro Yamazaki, Yu Sunakawa, Hiroya Taniguchi, Hideaki Bando, Manabu Shiozawa, Tomohiro Nishina, Hisateru Yasui, Yoshinori Kagawa, Naoki Takahashi, Tadamichi Denda, Taito Esaki, Hisato Kawakami, Hironaga Satake, Atsuo Takashima, Nobuhisa Matsuhashi, Takeshi Kato, Chiharu Asano, Yukiko Abe, Shogo Nomura, Takayuki Yoshino

**Affiliations:** ^1^ Department of Gastroenterology and Hepatology Hokkaido University Hospital Sapporo Japan; ^2^ Division of Gastrointestinal Oncology Shizuoka Cancer Center Nagaizumi Japan; ^3^ Department of Clinical Oncology St. Marianna University School of Medicine Kawasaki Japan; ^4^ Department of Clinical Oncology Aichi Cancer Center Hospital Nagoya Japan; ^5^ Department of Gastroenterology and Gastrointestinal Oncology National Cancer Center Hospital East Kashiwa Japan; ^6^ Department of Gastrointestinal Surgery Kanagawa Cancer Center Yokohama Japan; ^7^ Department of Gastrointestinal Medical Oncology National Hospital Organization Shikoku Cancer Center Matsuyama Japan; ^8^ Department of Medical Oncology Kobe City Medical Center General Hospital Kobe Japan; ^9^ Department of Surgery Kansai Rosai Hospital Amagasaki Japan; ^10^ Department of Gastroenterology Saitama Cancer Center Ina Japan; ^11^ Division of Gastroenterology Chiba Cancer Center Chiba Japan; ^12^ Department of Gastrointestinal and Medical Oncology National Hospital Organization Kyushu Cancer Center Fukuoka Japan; ^13^ Department of Medical Oncology Kindai University Faculty of Medicine Osaka‐sayama Japan; ^14^ Cancer Treatment Center Kansai Medical University Hospital Hirakata; ^15^ Department of Medical Oncology Kochi Medical School Nankoku Japan; ^16^ Department of Gastrointestinal Medical Oncology National Cancer Center Hospital Tokyo Japan; ^17^ Department of Gastroenterological Surgery and Pediatric Surgery Gifu University Hospital Gifu Japan; ^18^ Department of Surgery National Hospital Organization Osaka National Hospital Osaka Japan; ^19^ Institute of Health Science Innovation for Medical Care Hokkaido University Hospital Sapporo Japan; ^20^ G&G Science Co., Ltd. Fukushima Japan; ^21^ Department of Biostatistics and Bioinformatics, Graduate School of Medicine The University of Tokyo Tokyo Japan

**Keywords:** angiogenesis, biomarkers, chemotherapy, colorectal cancer

## Abstract

**Background:**

Several biomarkers have been established for metastatic colorectal cancer (mCRC). We investigated whether plasma angiogenesis factors could predict the efficacy of biologics combined with chemotherapy in first‐line (1L) treatment in patients with *RAS* wild‐type mCRC and the dynamics of plasma angiogenesis factors at progression during 1L treatment.

**Methods:**

In this multicenter prospective observational study, serial plasma samples were prospectively collected at pretreatment and progression stages; 17 plasma angiogenesis factors were analyzed using the multiplex assay with Luminex® technology. Interactions between the pretreatment measurements and treatment groups on progression‐free survival (PFS) and overall survival (OS) in patients with *RAS* wild‐type were assessed using the propensity‐score weighted Cox proportional hazards model.

**Results:**

From February 2018 to September 2020, 202 patients were enrolled in the 1L cohort; 133 patients had *RAS* wild‐type (chemotherapy plus bevacizumab [BEV group, *n* = 33] and plus anti‐epidermal growth factor receptor monoclonal antibodies [aEGFR group, *n* = 100]). A trend of strong interaction on PFS was observed for interleukin‐8 (IL‐8) (*p* = 0.0752) and soluble vascular cell adhesion molecule‐1 (sVCAM‐1) (*p* = 0.0156). Regarding OS, IL‐8 (*p* = 0.0283), soluble vascular endothelial growth factor‐receptor‐1 (sVEGFR‐1) (*p* = 0.0777) and sVCAM‐1 (*p* = 0.0011) tended to differentiate the treatment effect. In 112 patients, plasma samples were evaluable for dynamic analysis (57 and 55 from the BEV and aEGFR groups, respectively). In the BEV group, six factors significantly increased during progression, whereas two decreased. In the aEGFR group, three factors significantly increased, and six decreased.

**Conclusion:**

Pretreatment plasma IL‐8 and sVCAM‐1 levels could be predictive biomarkers to distinguish BEV and anti‐EGFR mAbs when combined with chemotherapy in the 1L treatment of *RAS* wild‐type mCRC. Several plasma angiogenesis factors showed significant change at progression in 1L chemotherapy plus biologics for *RAS* wild‐type mCRC, which are potential biomarkers for selecting an optimal angiogenesis inhibitor in second‐line treatment.

## 1 INTRODUCTION

Colorectal cancer (CRC) is the second leading cause of cancer‐related deaths worldwide, and metastatic or recurrent CRC has a poor prognosis.^1^ Therefore, the standard of care for patients with untreated unresectable metastatic CRC (mCRC) includes chemotherapy comprising cytotoxic agents with or without a targeted biologic agent of either bevacizumab (BEV) or anti‐epidermal growth factor receptor monoclonal antibodies (anti‐EGFR mAbs).^2^, ^3^, ^4^, ^5^ The latest guidelines recommend anti‐EGFR mAbs combined with doublet chemotherapy as the first‐line treatment (1L) for patients with left‐sided *RAS/BRAF* V600E wild‐type mCRC, according to a pooled meta‐analysis.^2^, ^3^, ^4^, ^5^, ^6^

It has been reported that plasma vascular endothelial growth factor D (VEGF‐D) and VEGF‐A are potential predictive biomarkers for antiangiogenic inhibitors in 1L and second‐line (2L) treatments. A plasma biomarker analysis of the CALGB80405 trial^7^ on 1L chemotherapy plus BEV versus chemotherapy plus cetuximab (CET) showed that patients with low serum VEGF‐D levels had significantly longer overall survival (OS) from chemotherapy plus BEV than CET (hazard ratio [HR] 0.62; 95% confidence interval [CI] 0.41–0.92). Additionally, the 1L MAX trial, which was a Phase III trial comparing capecitabine, capecitabine plus BEV (CB), and CB plus mitomycin (CBM), showed that patients with low VEGF‐D expression had significantly longer OS when treated with the BEV combined regimen (CB plus CBM vs. C) (HR 0.35; 95% CI: 0.13–0.90).^8^

In the 2L RAISE trial,^9^ which was a Phase III trial comparing fluorouracil, leucovorin, and irinotecan (FOLFIRI) plus ramucirumab (RAM) with FOLFIRI plus placebo, patients receiving RAM in the high VEGF‐D group (≥ 115 pg/mL) had a significantly longer survival than those receiving the placebo (HR 0.73; 95% CI: 0.60–0.89, *p* = 0.0022), while those with low VEGF‐D (<115 pg/mL) had shorter survival (HR 1.32; 95% CI: 1.02–1.70; *p* = 0.0344). Additionally, a significant interaction was observed between VEGF‐D levels (cut off, 115 pg/mL) and treatment arms (*p* = 0.0005). The WJOG6210G trial, which was a randomized Phase II trial comparing FOLFIRI plus panitumumab (PANI) and FOLFIRI plus BEV for patients with *RAS* wild‐type BEV refractory, demonstrated that FOLFIRI plus BEV had a better OS than FOLFIRI plus PANI in patients with low serum VEGF‐D (HR 0.82; 95% CI: 0.35–2.01)^10^ and those with high serum VEGF‐A (HR 0.67; 95% CI 0.48–1.69).^11^ However, the role of plasma angiogenesis‐related mediators as predictors of anti‐EGFR mAbs and angiogenesis inhibitors remains unknown.

Therefore, we aimed to investigate whether plasma angiogenesis factors could predict the efficacy of biologics combined with chemotherapy in 1L treatment in patients with *RAS* wild‐type mCRC and the dynamics of plasma angiogenesis factors at progression during 1L.

## 2 MATERIALS AND METHODS

### 2.1 Study design and participants

The SCRUM‐Japan GI‐SCREEN CRC‐Ukit trial was a multicenter prospective observational study investigating the association between plasma angiogenesis‐related factors and clinical outcomes in mCRC. Patients who met the following criteria were eligible for this trial: pathologically or histologically confirmed mCRC and *RAS* mutational status and age ≥20 years. In the 1L cohort, patients who had not been administered chemotherapy for metastatic disease were scheduled to receive chemotherapy plus either BEV or anti‐EGFR mAbs. In the 2L cohort, patients immediately after completion of chemotherapy plus either BEV or anti‐EGFR mAbs for the 1L treatment for mCRC were scheduled to receive FOLFIRI plus RAM or FOLFIRI plus aflibercept (AFL) or chemotherapy plus BEV. Longitudinal measurements of plasma angiogenesis‐related factors were analyzed using the multiplex assay with Luminex® technology. Here, we investigated whether plasma angiogenesis‐related mediators could predict the efficacy of biologics combined with chemotherapy as a 1L treatment in patients with *RAS* wild‐type mCRC and the dynamics of plasma angiogenesis‐related mediators during progression in 1L treatment.

A comparison of the efficacy parameters, such as response rate (RR), progression‐free survival (PFS), and OS of each biologic (1L chemotherapy plus BEV [BEV group] or 1L chemotherapy plus anti‐EGFR mAbs [aEGFR group]) depending on plasma angiogenesis‐related mediators were performed in patients with *RAS* wild‐type mCRC (efficacy analysis set). Additionally, analysis of dynamics in plasma angiogenesis‐related mediators was performed in patients with paired plasma samples (pretreatment and disease progression), regardless of *RAS* mutational status (dynamic analysis set). The analyses of the 2L cohort will be reported elsewhere in the future.

The Institutional Review Board at all participating centers reviewed and approved this study. This study was registered in the University Hospital Medical Information Network Clinical Trials Registry (UMIN000028616). This study was conducted following the Declaration of Helsinki. Written informed consent was obtained from all patients enrolled in this trial.

### 2.2 Data collection and assessments

The following clinical characteristics of the eligible patients were collected: age, sex, presence of primary tumor, *RAS/BRAF* mutational status, and microsatellite instability/mismatch repair (MSI/MMR) status. Regarding efficacies, antitumor effects of 1L treatment, PFS and OS, and RR were assessed. Tumor response assessment was conducted by treating physicians based on Response Evaluation Criteria in Solid Tumors ver1.1.^12^ Furthermore, PFS was defined as the time from the initiation of 1L chemotherapy to disease progression or death from any cause, whichever occurred first. OS was defined as the time from the initiation of 1L chemotherapy to death from any cause. Patients who were lost to follow‐up were censored at the date of the last response evaluation for PFS and the date of the last contact for OS.

### 2.3 Analysis of plasma angiogenesis‐related mediators

Plasma samples were collected twice in pairs from all patients pre‐ and post‐treatment. Overall, 17 plasma angiogenesis‐related mediators (hepatocyte growth factor [HGF], placental growth factor [PlGF], VEGF‐A, VEGF‐D, angiopoietin‐2, interferon‐γ, interleukin‐6 [IL‐6], IL‐8, soluble neuropilin‐1, thrombospondin‐2 [TSP‐2], osteopontin [OPN], soluble vascular endothelial growth factor‐receptor‐1 [sVEGFR1], sVEGFR2, sVEGFR3, soluble intercellular adhesion molecule‐1 [sICAM‐1], soluble vascular cell adhesion molecule‐1 [sVCAM‐1], and tissue inhibitor of metalloproteinase‐1 [TIMP‐1]) were analyzed using the multiplex assay using Luminex® technology.

First, angiogenesis factors from plasma samples were bound to their respective specific antibodies using Luminex® beads. Next, biotinylated detection antibodies were conjugated to antigen–antibody complex and labeled with streptavidin‐phycoerythrin. Subsequently, beads were analyzed using Luminex® 200, and fluorescent signals were calculated as concentrations from the standard curve for each substance. Finally, these angiogenesis factors were measured using Milliplex® assay kits (Merck).^13^, ^14^

### 2.4 Statistical analyses

A propensity‐score weighted Cox regression model was applied to screen out baseline plasma angiogenesis‐related mediators that would strongly interact with the treatment group (either BEV or aEGFR group) on PFS or OS. Continuous plasma angiogenesis variables were dichotomized according to their median values. The propensity score was estimated using a multivariable logistic regression model, including tumor location, an indicator of whether the number of combined cytotoxic agents was ≥3, their interaction term, sex, and age. Age was modeled as a natural cubic spline. In the propensity‐score weighted Cox regression model, the inverse of the estimated propensity score was used as a weight, and a robust sandwich estimator was used to calculate the standard error. The significance level for testing interaction terms was set at an alpha of 10%.

For baseline patient characteristics, categorical and continuous variables were compared using Fisher's exact and Wilcoxon rank‐sum tests, respectively. Additionally, survival curves were estimated and compared using the Kaplan–Meier method and log‐rank test, respectively.

## 3 RESULTS

### 3.1 Patients

**FIGURE 1:**
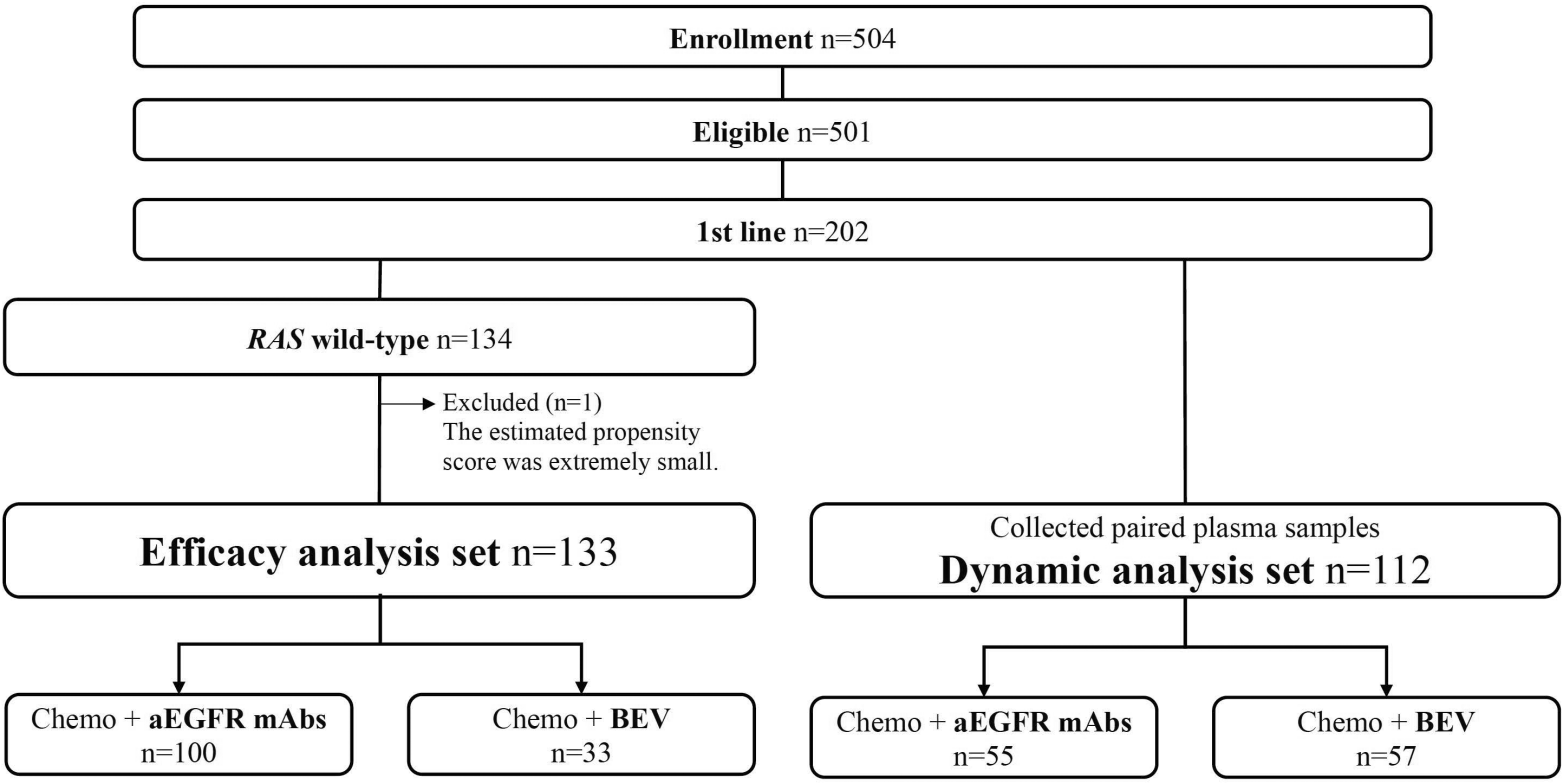
Flow diagram of this study. anti‐EGFR mAbs, anti‐epidermal growth factor receptor monoclonal antibodies; BEV, bevacizumab.

From February 2018 to September 2020, 504 patients with mCRC were enrolled at 27 institutions in Japan. Among the 501 eligible patients, 202 (40%) were enrolled as a 1L treatment cohort (Figure 1). In the efficacy analysis set, after excluding 68 patients with *RAS* mutations and one whose estimated propensity score was too small, 133 were included in the *RAS* wild‐type 1L cohort (BEV group: *n* = 33, aEGFR group: *n* = 100 [reference]).

Baseline characteristics of the *RAS* wild‐type 1L cohort are presented in Table 1. Several characteristics, including tumor location (*p* < 0.001), *BRAF* V600E mutational status (*p* < 0.001), MSI status (*p* = 0.002), and combined cytotoxic agents (*p* = 0.002), were imbalanced between the two groups. At the data cutoff date as of September 30, 2021, the median follow‐up time was significantly longer in the BEV group (35.7 months) than in the aEGFR group (28.8 months) (HR 0.436; 95% CI: 0.251–0.757, *p* = 0.002).

**TABLE 1:** Patient characteristics in the efficacy analysis set.

	All (*n* = 133)		aEGFR (*n* = 100)		BEV (*n* = 33)		*p*‐value
Age							
Median (range)	64 (26–85)		64 (26–84)		67 (45–85)		0.116
Sex							
Male	83	(62.4)	63	(63.0)	20	(60.6)	0.838
Female	50	(37.6)	37	(37.0)	13	(39.4)	
Tumor location							
Right	15	(11.3)	5	(5.0)	10	(30.3)	<0.001
Left	118	(88.7)	95	(95.0)	23	(69.7)	
*BRAF* ^a^							
Wild	115	(86.5)	94	(94.0)	21	(63.6)	<0.001
V600E mutant	12	(9.0)	2	(2.0)	10	(30.3)	
Unknown	6	(4.5)	4	(4.0)	2	(6.1)	
MSI^b^							
MSI‐high	4	(3.0)	0	(0)	4	(12.1)	0.002
Non‐MSI‐high	105	(78.9)	86	(86.0)	19	(57.6)	
Unknown	24	(18.1)	14	(14.0)	10	(30.3)	
Cytotoxic agents							
Doublet or mono	113	(85.0)	91	(91.0)	22	(66.7)	0.002
Triplet	20	(15.0)	9	(9.0)	11	(33.3)	

Abbreviations: aEGFR, anti‐epidermal growth factor receptor; BEV, bevacizumab; *BRAF*, v‐raf murine sarcoma viral oncogene homolog B1; MSI, microsatellite instability.

^a^ Six patients (aEGFR *n* = 4, BEV *n* = 2) had not undergone *BRAF* V600E analysis.

^b^ Twenty‐four patients (aEGFR *n* = 14, BEV *n* = 10) had not undergone MSI analysis.

### 3.2 PFS by baseline plasma angiogenesis‐related mediators

Median PFS in the efficacy analysis set was 13.9 (95% CI: 11.1–17.0) and 15.6 (95% CI 11.3‐not estimable [NE]) months in the aEGFR and BEV groups, respectively (HR 0.724; 95% CI: 0.434–1.207, *p* = 0.2140) (Figure S1A).

**FIGURE 2:**
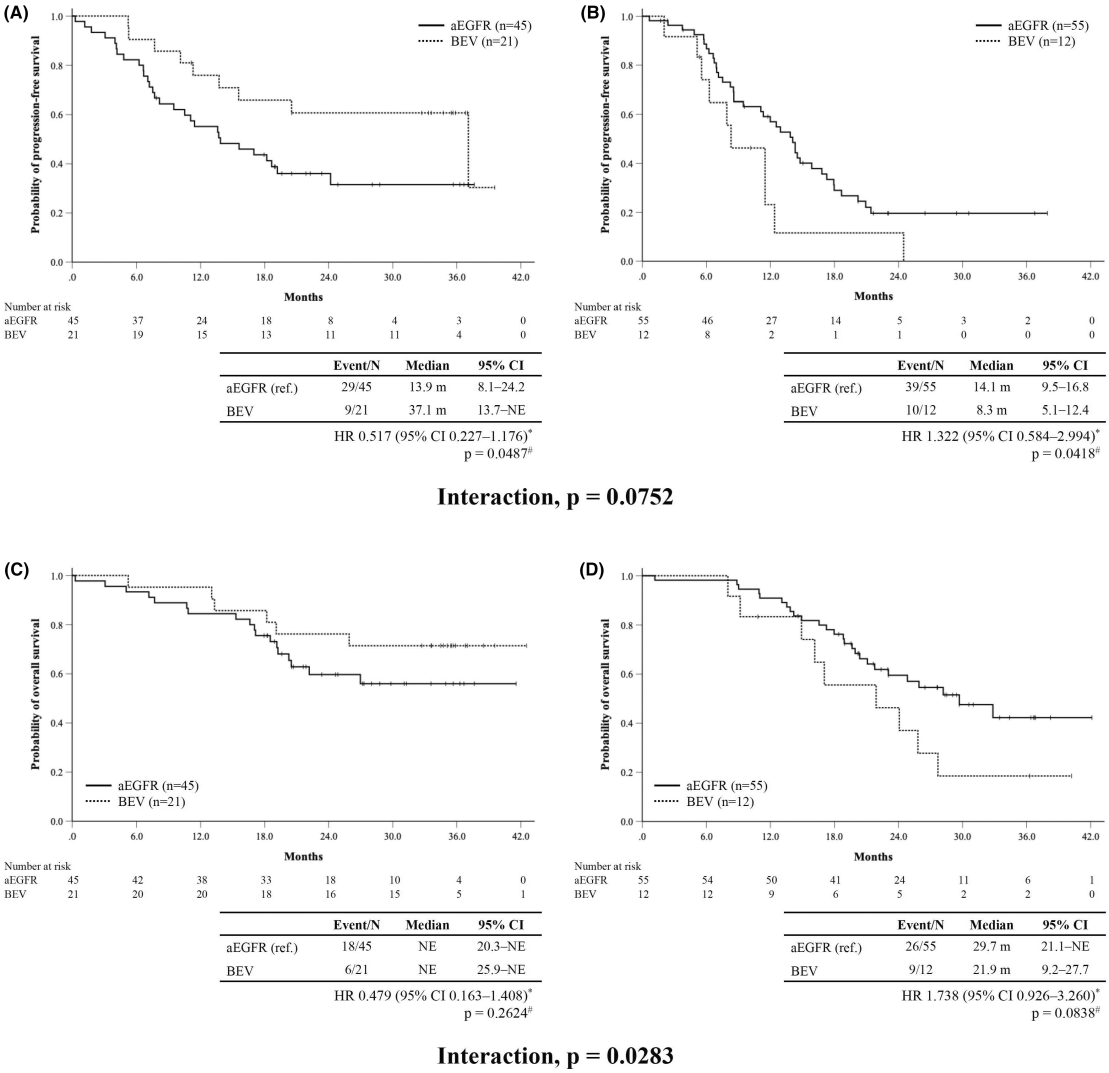
Progression‐free survival (PFS) and overall survival (OS) by IL‐8. Kaplan–Meier curves of the PFS in patients with below (A) and above (B) the median IL‐8. Kaplan–Meier curve of the OS in patients with below (C) and above (D) the median IL‐8. *p*‐values^#^ are calculated using the log‐rank test, two‐sided. HR* estimated by unstratified Cox model. aEGFR, anti‐epidermal growth factor receptor; BEV, bevacizumab; CI, confidence interval; HR, hazard ratio; IL‐8, interleukin 8; NE, not estimable.

**FIGURE 3:**
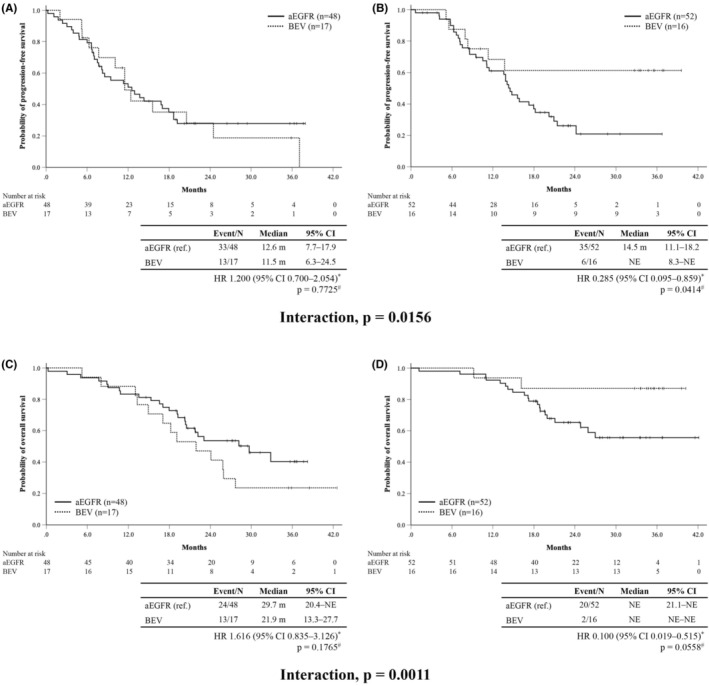
Progression‐free survival (PFS) and overall survival (OS) by sVCAM‐1. Kaplan–Meier curves of the PFS in patients with below (A) and above (B) the median sVCAM‐1. Kaplan–Meier curve of the OS in patients with below (C) and above (D) the median sVCAM‐1. *p*‐values^#^ are calculated using the log‐rank test, two‐sided. HR* estimated by unstratified Cox model. aEGFR, anti‐epidermal growth factor receptor; BEV, bevacizumab; CI, confidence interval; HR, hazard ratio; NE, not estimable; sVCAM‐1, soluble vascular cell adhesion molecule‐1.

The propensity‐score weighted Cox model for PFS is presented in Table 2. Significant interactions were found in IL‐8 (below median; aEGFR group 13.9 months vs. BEV group 37.1 months, HR 0.517 [95% CI: 0.227–1.176], *p* = 0.0487, above median: 14.1 months vs. 8.3 months, HR 1.322 [95% CI: 0.584–2.994], *p* = 0.0418, interaction *p* = 0.0752) (Figure 2A,B) and sVCAM‐1 (below median; aEGFR group 12.6 months vs. BEV group 11.5 months, HR 1.200 [95% CI: 0.700–2.054], *p* = 0.7725, above median: 14.5 months vs. NE, HR 0.285 [95% CI: 0.095–0.859], *p* = 0.0414, interaction *p* = 0.0156) (Figure 3A,B). Analyses according to quartiles and median values were performed, and these showed the same tendency those using only the median values in IL‐8 and sVCAM‐1 (Figures S2 and S3).

**TABLE 2:** Progression‐free survival by each baseline plasma angiogenesis factor in the efficacy analysis set.

	Below median					Above median					Interaction *p*
	*N*	Median (months)	95% CI	HR^a^ (95% CI)	*p*‐value*	*N*	Median (months)	95% CI	HR^a^ (95% CI)	*p*‐value*	
Angiopoietin‐2											
aEGFR	49	17.0	11.4–24.2	1	0.3436	51	12.0	7.1–14.3	1	0.2681	0.2772
BEV	16	NE	10.1–NE	0.871 (0.375–2.026)		17	12.4	7.7–37.1	0.407 (0.173–0.957)		
HGF											
aEGFR	49	15.9	11.1–NE	1	0.4959	51	12.9	8.5–15.6	1	0.2775	0.9103
BEV	17	NE	6.3–NE	0.703 (0.283–1.748)		16	12.4	7.9–37.1	0.572 (0.283–1.155)		
IL‐8											
aEGFR	45	13.9	8.1–24.2	1	0.0487	55	14.1	9.5–16.8	1	0.0418	0.0752
BEV	21	37.1	13.7–NE	0.517 (0.227–1.176)		12	8.3	5.1–12.4	1.322 (0.584–2.994)		
PlGF											
aEGFR	53	18.2	14.1–24.2	1	0.2160	47	8.5	6.7–12.6	1	0.2054	0.5847
BEV	13	NE	10.1–NE	0.662 (0.237–1.848)		20	12.4	6.3–24.5	0.464 (0.212–1.016)		
VEGF‐A											
aEGFR	47	18.2	11.4–NE	1	0.5029	53	12.0	7.5–14.3	1	0.3706	0.5050
BEV	18	20.5	11.3–NE	0.780 (0.356–1.713)		15	13.7	5.3–37.1	0.504 (0.193–1.315)		
VEGF‐D											
aEGFR	45	14.8	8.6–19.2	1	0.2005	55	12.6	8.6–17.0	1	0.9331	0.5283
BEV	21	24.5	11.3–37.1	0.554 (0.253–1.213)		12	11.5	5.6–NE	0.883 (0.377–2.071)		
OPN											
aEGFR	44	17.9	11.4–NE	1	0.4358	56	12.6	7.1–14.5	1	0.9301	0.6511
BEV	22	37.1	10.1–37.1	0.678 (0.284–1.620)		11	11.5	5.6–15.6	0.831 (0.433–1.597)		
sNeuropillin‐1											
aEGFR	49	18.0	12.0–NE	1	0.5487	51	11.4	8.5–14.3	1	0.3182	0.8627
BEV	17	37.1	7.9–37.1	0.693 (0.286–1.680)		16	11.5	7.7–NE	0.595 (0.279–1.267)		
sVEGFR‐1											
aEGFR	48	15.6	9.5–20.2	1	0.5621	52	12.6	8.6–15.9	1	0.3068	0.1751
BEV	18	13.7	11.3–NE	0.921 (0.449–1.888)		15	20.5	5.2–37.1	0.417 (0.159–1.093)		
sVEGFR‐2											
aEGFR	50	14.3	11.1–17.9	1	0.0898	50	13.9	8.2–18.7	1	0.7865	0.9991
BEV	16	24.5	11.3–37.1	0.543 (0.200–1.472)		17	12.4	5.6–NE	0.618 (0.296–1.290)		
sVEGFR‐3											
aEGFR	48	14.1	8.6–20.2	1	0.4288	52	13.9	9.5–15.9	1	0.3536	0.6643
BEV	18	15.6	11.3–NE	0.678 (0.335–1.372)		15	20.5	5.2–37.1	0.513 (0.185–1.419)		
TSP‐2											
aEGFR	48	18.7	13.7–NE	1	0.9561	52	11.1	7.7–14.1	1	0.0900	0.1061
BEV	18	15.6	11.3–NE	1.123 (0.506–2.490)		15	11.5	5.6–37.1	0.337 (0.126–0.904)		
sICAM‐1											
aEGFR	47	15.6	11.3–19.2	1	0.1926	53	12.6	8.5–15.9	1	0.9226	0.4979
BEV	19	37.1	11.3–NE	0.733 (0.351–1.533)		14	11.5	5.2–NE	0.527 (0.194–1.432)		
sVCAM‐1											
aEGFR	48	12.6	7.7–17.9	1	0.7725	52	14.5	11.1–18.2	1	0.0414	0.0156
BEV	17	11.5	6.3–24.5	1.200 (0.700–2.054)		16	NE	8.3–NE	0.285 (0.095–0.859)		
TIMP‐1											
aEGFR	45	18.7	11.4–NE	1	0.9288	55	12.0	8.5–14.3	1	0.1916	0.4464
BEV	21	20.5	10.1–37.1	0.885 (0.395–1.982)		12	11.5	6.3–NE	0.516 (0.213–1.250)		

*Note*: For interferon‐γ and IL‐6, more than half of the patients were below the lower detection limit (2.00 pg/mL), and no analysis was performed.

Abbreviations: aEGFR, anti‐epidermal growth factor receptor; BEV, bevacizumab; CI, confidence interval; HGF, hepatocyte growth factor; HR, hazard ratio; IL, interleukin; NE, not‐estimable; OPN, osteopontin; PlGF, placental growth factor; sICAM‐1, soluble intercellular adhesion molecule‐1; sVCAM‐1, soluble vascular cell adhesion molecule‐1; sVEGFR, soluble vascular endothelial growth factor‐receptor; TIMP‐1, tissue inhibitor of metalloproteinase‐1; TSP‐2, thrombospondin‐2; VEGF, vascular endothelial growth factor.

^a^ Estimated by IPTW Cox model.

* Log‐rank test, two‐sided.

### 3.3 OS by baseline plasma angiogenesis‐related mediators

Median OS in the efficacy analysis set was 32.9 months (95% CI: 23.1 months–NE) in the aEGFR group and not reached (95% CI: 19.1 months–NE) in the BEV group (HR 0.908; 95% CI: 0.504–1.637, *p* = 0.7468) (Figure S1B). The propensity‐score weighted Cox model for OS is presented in Table 3. Significant interactions were observed in IL‐8 (below median; aEGFR group NE vs. BEV group NE, HR 0.479 [95% CI: 0.163–1.408], *p* = 0.2624, above median: 29.7 months vs. 21.9 months, HR 1.738 [95% CI: 0.926–3.260], *p* = 0.0838, interaction *p* = 0.0283) (Figure 2C,D), sVEGFR‐1 (below median; aEGFR group NE vs. BEV group 25.9 months, HR 1.311 [95% CI: 0.565–3.041], *p* = 0.3004, above median: 25.9 months vs. NE, HR 0.333 [95% CI: 0.091–1.215], *p* = 0.1770, interaction *p* = 0.0777), and sVCAM‐1 (below median; aEGFR group 29.7 months vs. BEV group 21.9 months, HR 1.616 [95% CI 0.835–3.126], *p* = 0.1765, above median: NE vs. NE, HR 0.100 [95% CI: 0.019–0.515], *p* = 0.0558, interaction *p* = 0.0011) (Figure 3C,D). Analyses according to quartiles and median values were also performed for OS, and these trends were similar to those of PFS (Figures S4 and S5).

**TABLE 3:** Overall survival by each baseline plasma angiogenesis factor in the efficacy analysis set.

	Below median					Above median					Interaction *p*
	*N*	PFS (months)	95% CI	HR^a^ (95% CI)	*p*‐value*	*N*	PFS (months)	95% CI	HR^a^ (95% CI)	*p*‐value*	
Angiopoietin‐2											
aEGFR	49	NE	25.9–NE	1	0.7542	51	24.8	19.6–NE	1	0.8159	0.2030
BEV	16	NE	14.9–NE	0.847 (0.281–2.560)		17	25.9	17.1–NE	0.577 (0.237–1.406)		
HGF											
aEGFR	49	NE	22.2–NE	1	0.6766	51	25.9	19.9–NE	1	0.9570	0.6994
BEV	17	NE	18.2–NE	0.683 (0.221–2.113)		16	25.8	16.2–NE	0.810 (0.379–1.729)		
IL‐8											
aEGFR	45	NE	20.3–NE	1	0.2624	55	29.7	21.1–NE	1	0.0838	0.0283
BEV	21	NE	25.9–NE	0.479 (0.163–1.408)		12	21.9	9.2–27.7	1.738 (0.926–3.260)		
PlGF											
aEGFR	53	NE	25.9–NE	1	0.3476	47	21.8	18.5–NE	1	0.9312	0.6822
BEV	13	NE	25.9–NE	0.523 (0.125–2.184)		20	24.1	14.9–NE	0.701 (0.308–1.594)		
VEGF‐A											
aEGFR	47	NE	NE–NE	1	0.7035	53	24.8	19.3–32.9	1	0.6171	0.4504
BEV	18	NE	19.1–NE	1.001 (0.369–2.718)		15	27.7	13.0–NE	0.574 (0.214–1.535)		
VEGF‐D											
aEGFR	45	NE	23.1–NE	1	0.5119	55	27.0	19.9–NE	1	0.3900	0.3017
BEV	21	NE	25.8–NE	0.597 (0.210–1.700)		12	23.0	9.2–NE	1.112 (0.496–2.492)		
OPN											
aEGFR	44	NE	23.1–NE	1	0.5475	56	25.9	18.8–NE	1	0.3740	0.1122
BEV	22	NE	18.2–NE	0.514 (0.159–1.661)		11	24.1	14.9–25.9	1.372 (0.778–2.420)		
sNeuropillin‐1											
aEGFR	49	NE	27.0–NE	1	0.6966	51	23.1	19.3–NE	1	0.9480	0.3727
BEV	17	NE	13.3–NE	0.517 (0.150–1.782)		16	25.8	17.1–NE	0.901 (0.431–1.883)		
sVEGFR‐1											
aEGFR	48	NE	27.0–NE	1	0.3004	52	25.9	20.5–NE	1	0.1770	0.0777
BEV	18	25.9	17.1–NE	1.311 (0.565–3.041)		15	NE	9.2–NE	0.333 (0.091–1.215)		
sVEGFR‐2											
aEGFR	50	32.9	19.9–NE	1	0.4801	50	NE	21.8–NE	1	0.7826	0.7166
BEV	16	NE	17.1–NE	0.565 (0.201–1.589)		17	27.7	16.2–NE	0.787 (0.326–1.900)		
sVEGFR‐3											
aEGFR	48	NE	19.9–NE	1	0.9739	52	28.2	21.8–NE	1	0.6086	0.8129
BEV	18	NE	19.1–NE	0.717 (0.314–1.639)		15	NE	13.3–NE	0.612 (0.177–2.112)		
TSP‐2											
aEGFR	48	NE	NE–NE	1	0.3035	52	23.1	19.6–32.9	1	0.2759	0.1413
BEV	18	NE	18.2–NE	1.396 (0.528–3.695)		15	NE	16.2–NE	0.413 (0.158–1.080)		
sICAM‐1											
aEGFR	47	NE	25.9–NE	1	0.7873	53	29.7	19.9–NE	1	0.8593	0.5703
BEV	19	NE	24.1–NE	0.837 (0.346–2.027)		14	19.1	9.2–NE	0.612 (0.201–1.858)		
sVCAM‐1											
aEGFR	48	29.7	20.4–NE	1	0.1765	52	NE	21.1–NE	1	0.0558	0.0011
BEV	17	21.9	13.3–27.7	1.616 (0.835–3.126)		16	NE	NE–NE	0.100 (0.019–0.515)		
TIMP‐1											
aEGFR	45	NE	27.0–NE	1	0.7395	55	24.8	19.9–NE	1	0.9116	0.7593
BEV	21	NE	19.1–NE	0.801 (0.284–2.259)		12	25.8	14.9–NE	0.860 (0.390–1.895)		

*Note*: For interferon‐γ and IL‐6, more than half of the patients were below the lower detection limit (2.00 pg/mL), and no analysis was performed.

Abbreviations: aEGFR, anti‐epidermal growth factor receptor; BEV, bevacizumab; CI, confidence interval; HGF, hepatocyte growth factor; HR, hazard ratio; IL, interleukin; NE, not‐estimable; OPN, osteopontin; PFS, progression‐free survival; PlGF, placental growth factor; sICAM‐1, soluble intercellular adhesion molecule‐1; sVCAM‐1, soluble vascular cell adhesion molecule‐1; sVEGFR, soluble vascular endothelial growth factor‐receptor; TIMP‐1, tissue inhibitor of metalloproteinase‐1; TSP‐2, thrombospondin‐2; VEGF, vascular endothelial growth factor.

^a^ Estimated by IPTW Cox model.

* Log‐rank test, two‐sided.

### 3.4 RR by baseline plasma angiogenesis‐related mediators

The RR in the efficacy analysis set was 74.2% and 48.4% in the aEGFR and BEV groups, respectively, significantly superior to that in the aEGFR group (odds ratio [OR] 0.326; 95% CI: 0.141–0.753, *p* = 0.014) (Table S1).

The RR showed significant interactions in IL‐8 (below median: OR 0.431, *p* = 0.169, above median: OR 0.280, *p* = 0.111, interaction *p* = 0.086), PlGF (below median: OR 0.385, *p* = 0.162, above median: OR 0.318, *p* = 0.052, interaction *p* = 0.019), sNeuropillin‐1 (below median: OR 0.301, *p* = 0.092, above median: OR 0.343, *p* = 0.080, interaction *p* = 0.057), sVEGFR‐1 (below median: OR 0.256, *p* = 0.050, above median: OR 0.322, *p* = 0.111, interaction *p* = 0.016), sVEGFR‐2 (below median: OR 0.264, *p* = 0.043, above median: OR 0.377, *p* = 0.139, interaction *p* = 0.031), and sVEGFR‐3 (below median: OR 0.397, *p* = 0.199, above median: OR 0.231, *p* = 0.027, interaction *p* = 0.034) (Table S2).

### 3.5 Dynamics of plasma angiogenesis‐related mediators in pretreatment and at progression

In the dynamics analysis set, after excluding 90 patients due to the ongoing treatment, discontinuation without progression, and lack of sample collection at progression, 112 were analyzed using the dynamics of plasma angiogenesis‐related mediators (BEV group: *n* = 57, aEGFR group: *n* = 55) (Figure 1).

Baseline plasma angiogenesis‐related mediators in the efficacy analysis set are presented in Table S3. IL‐6, IL‐8, OPN, and TIMP‐1 were significantly higher in the aEGFR group than in the BEV group.

In the aEGFR group, at progression, HGF (*p* = 0.035), VEGF‐D (*p* = 0.002), and sVCAM‐1 (*p* = 0.021) were significantly higher than those at pretreatment, whereas IL‐8 (*p* = 0.049), VEGF‐A (*p* = 0.007), sVEGFR1 (*p* < 0.001), sVEGFR3 (*p* = 0.002), TSP‐2 (*p* = 0.003), and TIMP‐1 (*p* = 0.023) were significantly lower. However, HGF (*p* = 0.019), PlGF (*p* < 0.001), VEGF‐D (*p* = 0.021), OPN (*p* < 0.001), sICAM‐1 (*p* < 0.001), and sVCAM‐1 (*p* < 0.001) were significantly higher at progression, whereas VEGF‐A (*p* < 0.001) and sVEGFR2 (*p* = 0.006) were lower in the BEV group (Table 4).

**TABLE 4:** Dynamics of plasma angiogenesis‐related factors at pretreatment and disease progression in the dynamic analysis set.

Median (pg/mL)	aEGFR (*n* = 55)				BEV (*n* = 57)			
	Pretreatment	At progression		*p*‐value	Pretreatment	At progression		*p*‐value
Angiopoietin‐2	1580	1470		0.132	1450	1270		0.092
HGF	131	158	Increase	0.035	129	168	Increase	0.019
IFN‐γ	<2.00	<2.00		0.276	<2.00	<2.00		0.248
IL‐6	<2.00	<2.00		0.350	<2.00	<2.00		0.112
IL‐8	12.2	8.22	Decrease	0.049	8.03	8.63		0.383
PlGF	7.11	6.26		0.384	7.31	21.5	Increase	<0.001
VEGF‐A	182	102	Decrease	0.007	97.1	<30.0	Decrease	<0.001
VEGF‐D	309	451	Increase	0.002	291	375	Increase	0.021
OPN	16,300	17,100		0.194	10,800	18,400	Increase	<0.001
sNeuropillin‐1	321,000	368,000		0.090	317,000	313,000		0.385
sVEGFR‐1	1490	703	Decrease	<0.001	1090	1220		0.314
sVEGFR‐2	11,100	9530		0.131	11,000	9610	Decrease	0.006
sVEGFR‐3	22,900	14,600	Decrease	0.002	17,200	20,000		0.411
TSP‐2	28,900	14,200	Decrease	0.003	19,600	19,000		0.481
sICAM‐1	567,000	419,000		0.371	261,000	398,000	Increase	<0.001
sVCAM‐1	1,040,000	1,120,000	Increase	0.021	956,000	1,250,000	Increase	<0.001
TIMP‐1	439,000	310,000	Decrease	0.023	292,000	281,000		0.460

Abbreviations: aEGFR, anti‐epidermal growth factor receptor; BEV, bevacizumab; HGF, hepatocyte growth factor; IFN‐γ, interferon‐γ; IL, interleukin; OPN, osteopontin; PlGF, placental growth factor; sICAM‐1, soluble intercellular adhesion molecule‐1; sVCAM‐1, soluble vascular cell adhesion molecule‐1; sVEGFR, soluble vascular endothelial growth factor‐receptor; TIMP‐1, tissue inhibitor of metalloproteinase‐1; TSP‐2, thrombospondin‐2; VEGF, vascular endothelial growth factor.

## 4 DISCUSSION

We demonstrated that baseline plasma IL‐8 and sVCAM‐1 levels could be biomarkers that distinguish between BEV and anti‐EGFR mAbs combined with cytotoxic agents in 1L treatment for patients with *RAS* wild‐type mCRC. Furthermore, in the analysis of dynamics for angiogenesis‐related mediators during pre‐ and post‐1L treatment, several angiogenesis‐related mediators were significantly changed during progression. These mediators may become biomarkers for selecting an optimal angiogenesis inhibitor in 2L treatment for patients with *RAS* wild‐type mCRC.

A pro‐inflammatory cytokine, IL‐8, alternatively known as CXCL8,^15^ plays a significant role in tumor growth, angiogenesis (formation of new blood vessels), and immune cell recruitment.^16^ BEV is a monoclonal antibody that targets VEGF, a key signaling molecule involved in angiogenesis.^17^ It inhibits VEGF, thereby reducing the formation of new blood vessels in tumors. IL‐8 promotes angiogenesis by stimulating endothelial cell migration and proliferation.^16^ In the context of anti‐angiogenic inhibitors, high levels of IL‐8 may contribute to resistance to the drug's anti‐angiogenic effects. Tumors with elevated IL‐8 expression may have increased vascularization and are more likely to continue growing despite anti‐angiogenic inhibitor. In our study, IL‐8 showed a significant interaction with PFS and OS, and low IL‐8 demonstrated favorable PFS and OS in the BEV group. High IL‐8 serum levels and tumor expression are associated with poor prognosis across many malignant diseases.^18^, ^19^, ^20^, ^21^ Correlations between IL‐8 and angiogenesis inhibitors have been reported in other cancers. From hepatocellular carcinoma and renal cell carcinoma analyses, higher baseline plasma levels of IL‐8 were correlated with worse outcomes in treatment with angiogenesis inhibitors, such as sunitinib or BEV. These results support our analysis, and IL‐8 levels may predict clinical response to antiangiogenic agents.^22^, ^23^, ^24^ However, a few reports exist on the significance of baseline IL‐8 in BEV combination chemotherapy for mCRC; thus, further investigation is needed.

VCAM‐1 is a Type‐1 transmembrane glycoprotein essential for cell adhesion needed for metastasis and is expressed on endothelial cells in response to VEGF stimulation or inflammation.^25^ VCAM‐1 is soluble and may function as an attractant for endothelial cells.^26^ Evidence from various cancer types suggests that VCAM‐1 is associated with a poor outcome.^27^, ^28^, ^29^, ^30^ In our analysis, sVCAM‐1 showed a significant interaction with PFS and OS, and high sVCAM‐1 demonstrated favorable PFS and OS in the BEV group. In a previous study, a baseline high level of sVCAM‐1 was significantly correlated with shorter OS in patients with mCRC receiving capecitabine, oxaliplatin, and BEV,^21^ of which results were inconsistent with our findings. However, Liu et al. reported that higher baseline levels of VCAM‐1 appeared to predict PFS (interaction *p* = 0.022) and OS (interaction *p* = 0.012) benefits from chemotherapy plus regorafenib over that plus placebo,^31^ which supports our findings of sVCAM‐1 as the potential predictive factor for BEV.

In our analysis, VEGF‐A was decreased and VEGF‐D was significantly increased at progression in the aEGFR group. In contrast, VEGF‐A was decreased, and both PlGF and VEGF‐D were significantly increased at progression in the BEV group. The dynamics in VEGF‐A, VEGF‐D, and PlGF after BEV administration, regardless of the primary organ, have been reported,^32^, ^33^, ^34^ and we found consistent results in our analysis. Although angiogenesis inhibitors, such as BEV, RAM, and AFL, are key drugs used in 2L, no clear selection criteria are available. Given the results of our analysis, using the RAM for the 1L patients with anti‐EGFR mAbs refractory and using either RAM or AFL in the 1L BEV refractory patients might be theoretically effective options because each drug inhibits VEGF‐D and PlGF. Therefore, we plan to analyze the relationship between the dynamics of plasma angiogenesis‐related factors and clinical outcome using a 2L cohort. We aim to present this aspect in another study.

VEGF‐A and VEGF‐D values may be potential predictive biomarkers for BEV.^7^, ^8^, ^10^, ^11^ In our study, the aEGFR group tended to have better PFS and OS outcomes in patients with high VEGF‐A levels (HR 0.504 and 0.574, respectively). In contrast, the BEV treatment tended to result in better survival outcomes in patients with low VEGF‐D levels (HR 0.554/0.597) without significant interaction. However, the role of VEGF‐A and VEGF‐D as predictors for anti‐EGFR mAbs and BEV remains controversial. Therefore, it warrants further investigation.

This study had some limitations. First, this was an observational study with a small number of patients with *RAS* wild‐type. Second, because the treatment regimen was decided by the physician's choice, some significant background differences were observed, mainly for the primary tumor location, *BRAF* status, MSI status, and combined cytotoxic agents (doublet/mono or triplet), which reflect the present standard‐of‐care in the world. Therefore, in the future, we plan to conduct validation trials and basic research using cell lines to verify the results of this study.

In conclusion, pretreatment plasma IL‐8 and sVCAM‐1 levels could be predictive biomarkers to distinguish between BEV and aEGFR when combined with chemotherapy in the 1L treatment for *RAS* wild‐type mCRC; thus, further investigation is warranted. Additionally, several plasma angiogenesis factors significantly changed during progression, implying that they might become biomarkers for selecting an optimal angiogenesis inhibitor in 2L treatment.

## AUTHOR CONTRIBUTIONS

**Satoshi Yuki:** Conceptualization (lead); data curation (equal); investigation (equal); methodology (lead); project administration (lead); supervision (lead); writing – original draft (lead); writing – review and editing (equal). **Kentaro Yamazaki:** Conceptualization (equal); data curation (equal); investigation (equal); methodology (equal); writing – review and editing (equal). **Yu Sunakawa:** Conceptualization (equal); data curation (equal); investigation (equal); methodology (equal); writing – review and editing (equal). **Hiroya Taniguchi:** Conceptualization (equal); data curation (equal); investigation (equal); methodology (equal); writing – review and editing (equal). **Hideaki Bando:** Conceptualization (equal); data curation (equal); investigation (equal); methodology (equal); writing – review and editing (equal). **Manabu Shiozawa:** Data curation (equal); investigation (equal); writing – review and editing (equal). **Tomohiro Nishina:** Data curation (equal); investigation (equal); writing – review and editing (equal). **Hisateru Yasui:** Data curation (equal); investigation (equal); writing – review and editing (equal). **Yoshinori Kagawa:** Data curation (equal); investigation (equal); writing – review and editing (equal). **Naoki Takahashi:** Data curation (equal); investigation (equal); writing – review and editing (equal). **Tadamichi Denda:** Data curation (equal); investigation (equal); writing – review and editing (equal). **Taito Esaki:** Data curation (equal); investigation (equal); writing – review and editing (equal). **Hisato Kawakami:** Data curation (equal); investigation (equal); writing – review and editing (equal). **Hironaga Satake:** Data curation (equal); investigation (equal); writing – review and editing (equal). **Atsuo Takashima:** Data curation (equal); investigation (equal); writing – review and editing (equal). **Nobuhisa Matsuhashi:** Data curation (equal); investigation (equal); writing – review and editing (equal). **Takeshi Kato:** Data curation (equal); investigation (equal); writing – review and editing (equal). **Chiharu Asano:** Data curation (lead); writing – review and editing (equal). **Yukiko Abe:** Methodology (equal); writing – review and editing (equal). **Shogo Nomura:** Formal analysis (lead); investigation (equal); methodology (equal); writing – review and editing (equal). **Takayuki Yoshino:** Conceptualization (equal); funding acquisition (lead); investigation (equal); methodology (equal); project administration (equal); supervision (equal); writing – review and editing (equal).

## CONFLICT OF INTEREST STATEMENT

Kentaro Yamazaki received lecture fees from Chugai Pharmaceutical Co., Ltd. (Chugai) and Takeda Pharmaceutical Co., Ltd. (Takeda). Yu Sunakawa received lecture fees from Chugai, Takeda, and Merck Biopharma Co., Ltd. (Merck Biopharma). Hiroya Taniguchi received a research grant from Takeda; received lecture fees from Chugai and Takeda. Yoshinori Kagawa received lecture fees from Chugai, Takeda, and Merck Biopharma. Taito Esaki received a research grant from Chugai; received lecture fees from Chugai. Hironaga Satake received lecture fees from Chugai, Takeda, and Merck Biopharma. Takeshi Kato received lecture fees from Takeda. Yukiko Abe is a Board Member of G&G SCIENCE Co., Ltd. Takayuki Yoshino received a research grant from Chugai; received lecture fees from Chugai and Merck Biopharma. Satoshi Yuki, Hideaki Bando, Manabu Shiozawa, Tomohiro Nishina, Hisateru Yasui, Naoki Takahashi, Tadamichi Denda, Hisato Kawakami, Atsuo Takashima, Nobuhisa Matsuhashi, Shogo Nomura, and Chiharu Asano have no conflict of interest.

## FUNDING INFORMATION

This study was supported by a grant from the Japan Agency for Medical Research and Development (17ck0106233h0002 to Takayuki Yoshino).

## ETHICS STATEMENT

This study was approved by the Institutional Review Board of each participating institution and was conducted in accordance with the Declaration of Helsinki and the Japanese Ethical Guidelines for Medical and Health Research Involving Human Subjects.

## Supporting information


Figure S1:

Figure S2:

Figure S3:

Figure S4:

Figure S5:

Table S1.

## Data Availability

To protect the privacy and confidentiality of patients in this study, clinical data are not made publicly available in a repository or the supplementary material of the article but will be made available following reasonable request to the corresponding author.
