# Supplementary figures and images for: Role of plasma angiogenesis factors in the efficacy of first‐line chemotherapy combined with biologics in RAS wild‐type metastatic colorectal cancer: Results from the GI‐SCREEN CRC‐Ukit study
Source: Cancer Med. 2023 Aug 28;12(18):18702–16. doi: 10.1002/cam4.6486 (PMC10557901; doi:10.1002/cam4.6486)

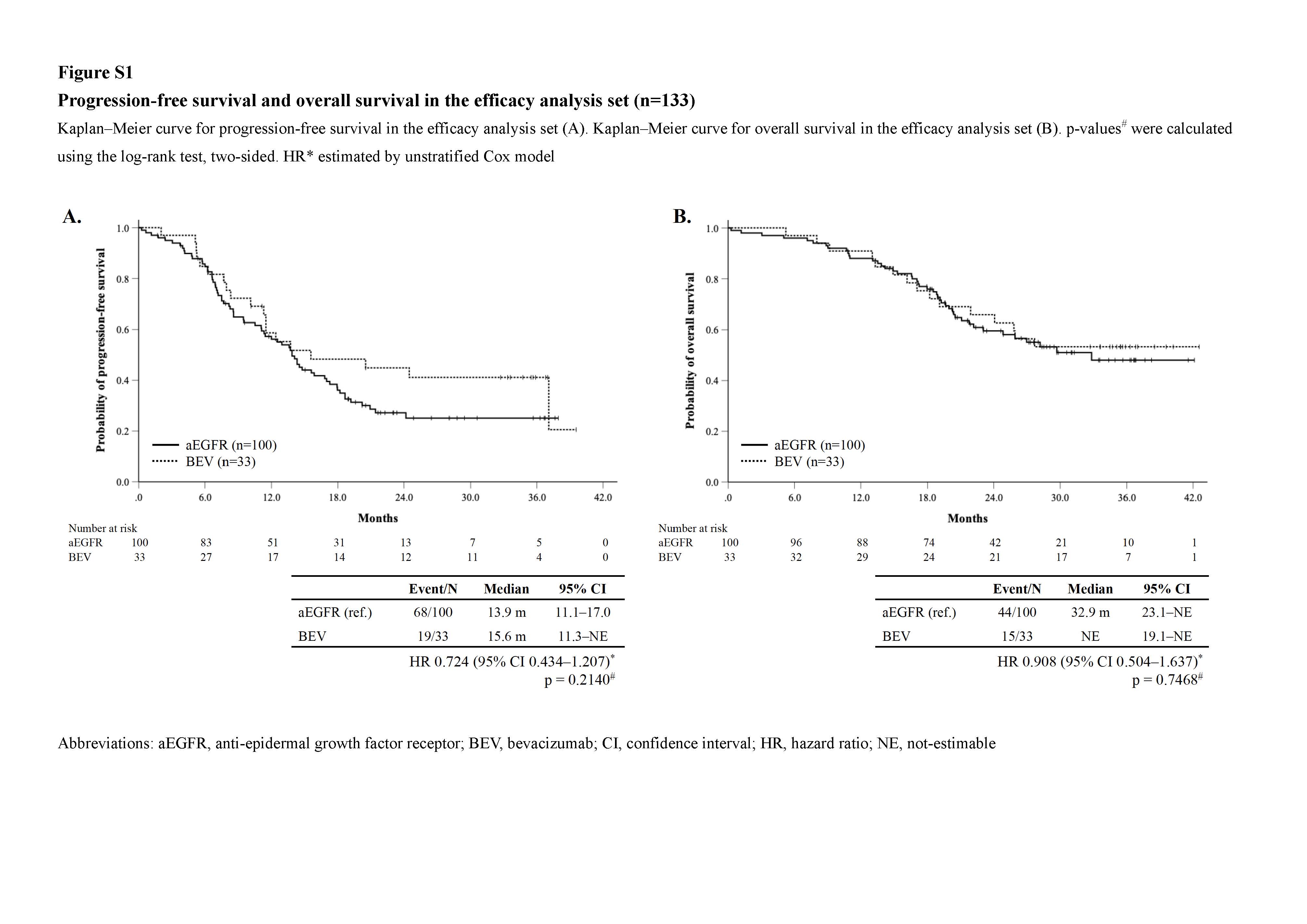

Supplement: Supplementary file 1 — Figure S1: [file CAM4-12-18702-s001.jpg]

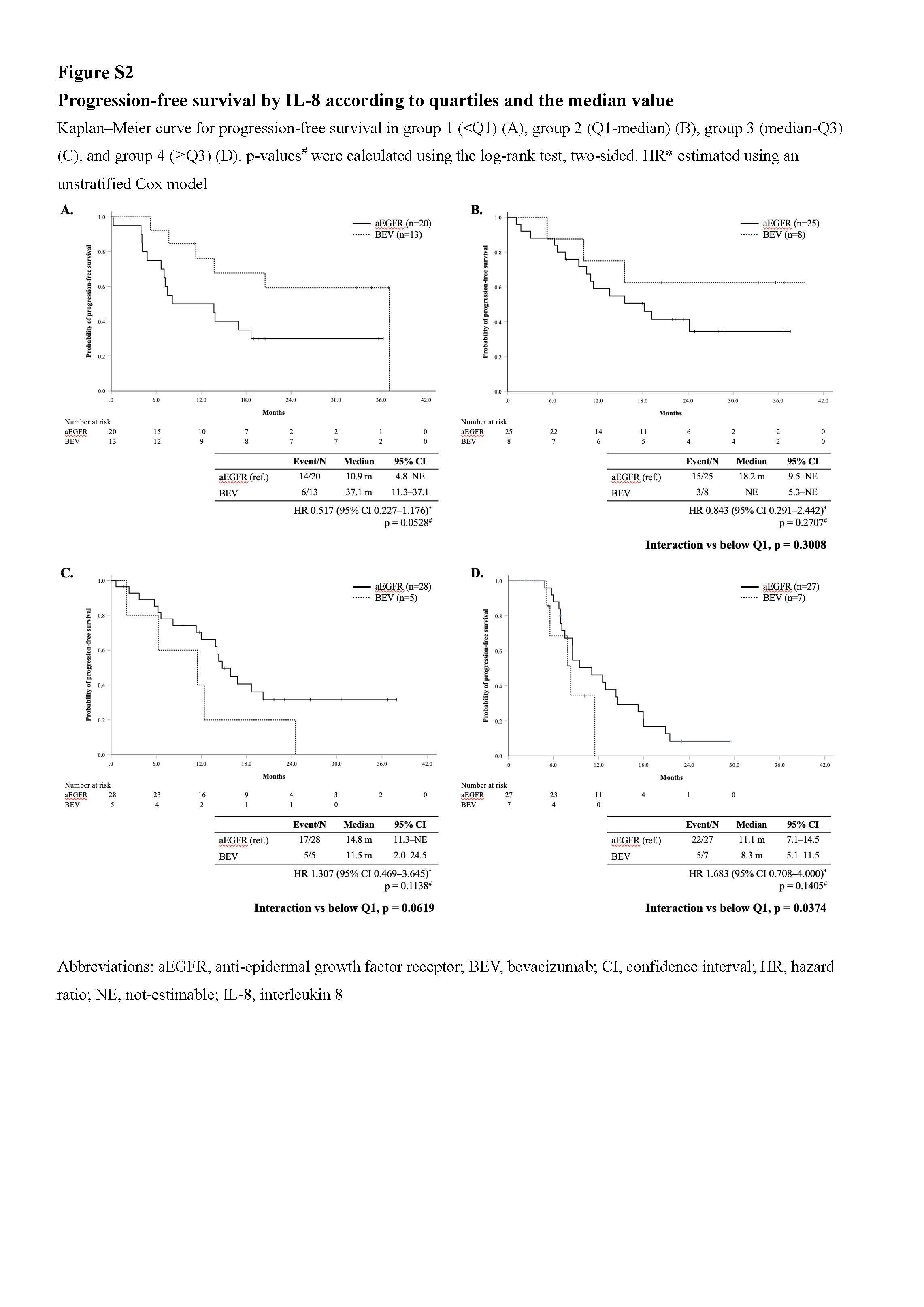

Supplement: Supplementary file 2 — Figure S2: [file CAM4-12-18702-s006.jpg]

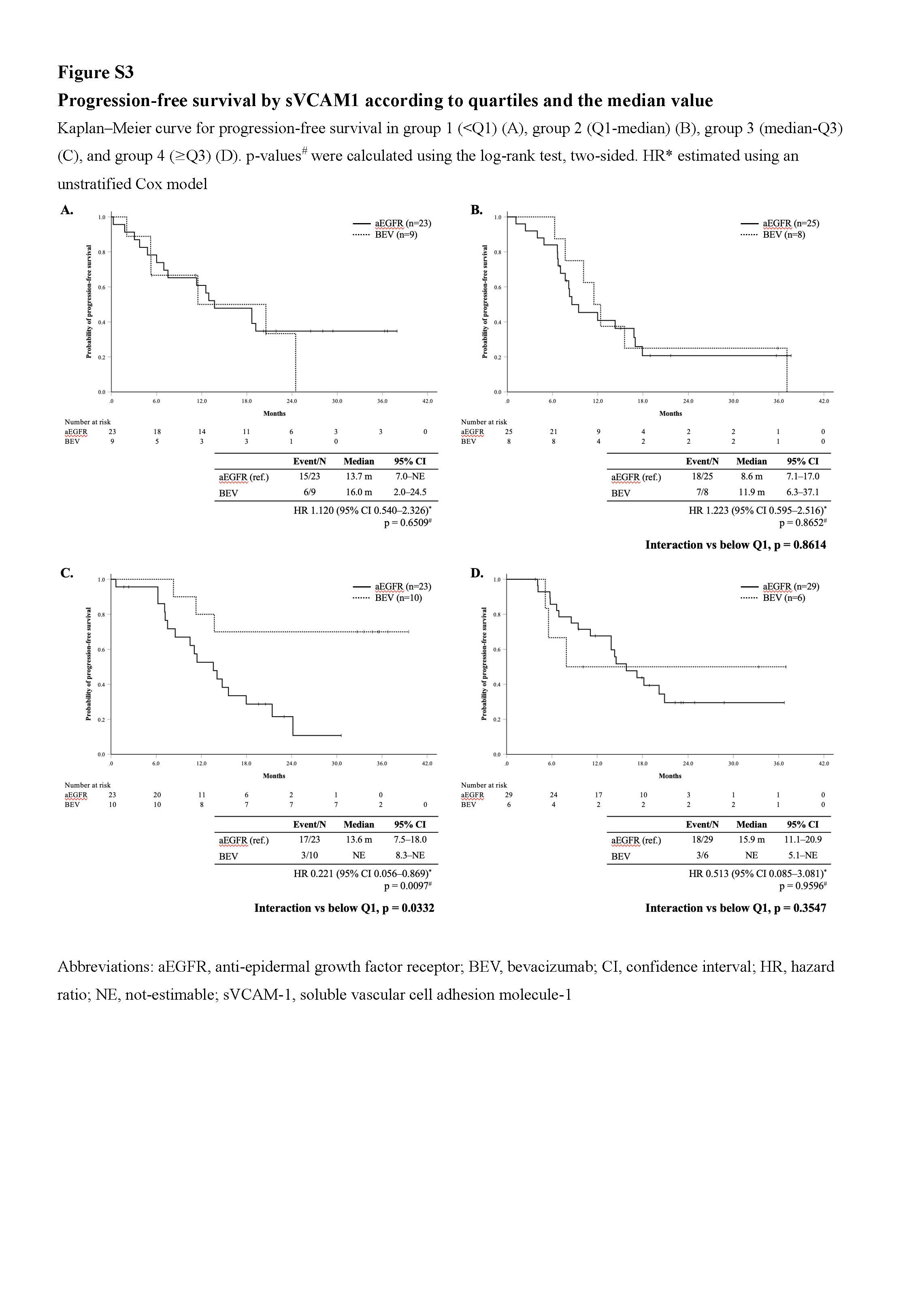

Supplement: Supplementary file 3 — Figure S3: [file CAM4-12-18702-s003.jpg]

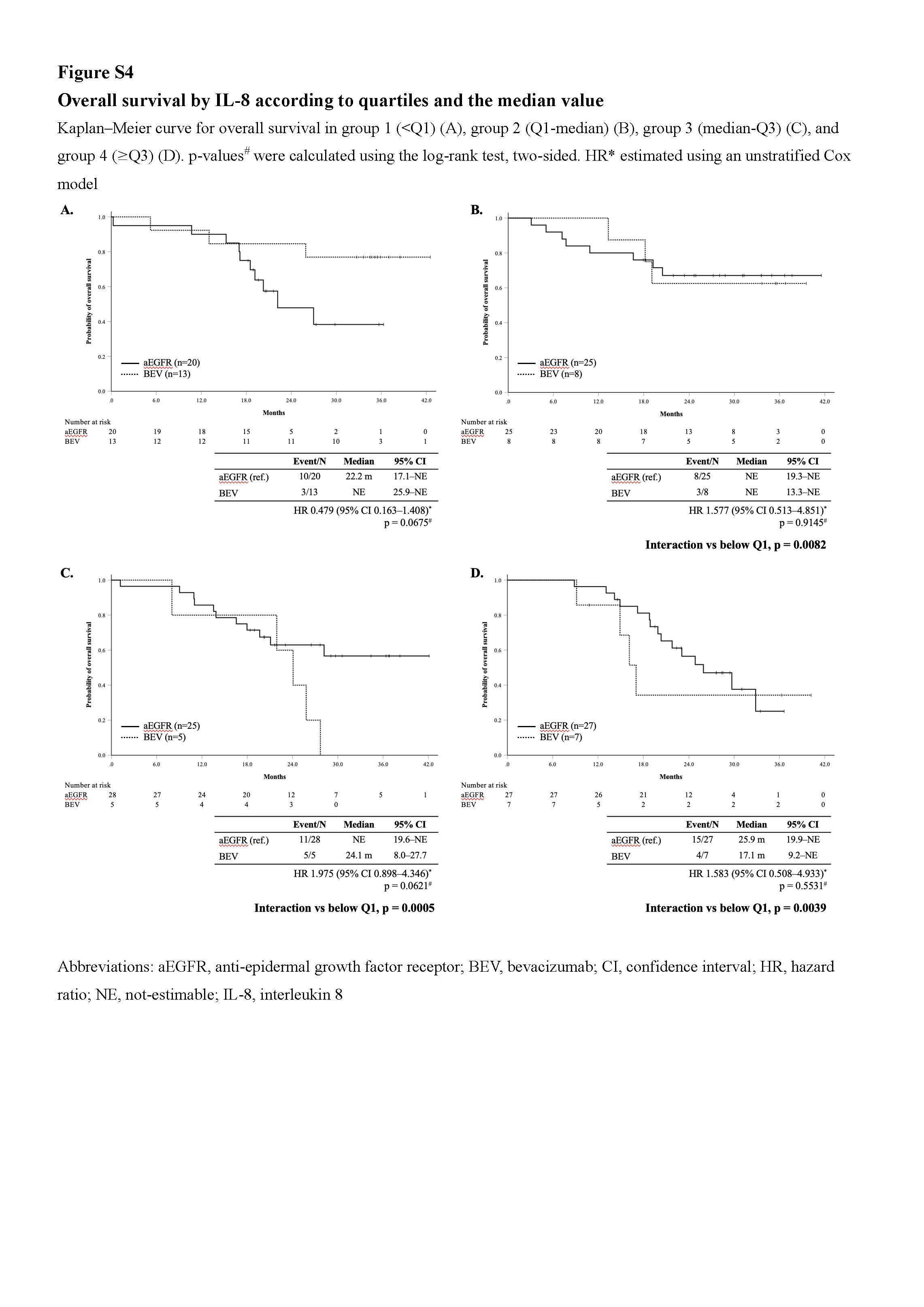

Supplement: Supplementary file 4 — Figure S4: [file CAM4-12-18702-s004.jpg]

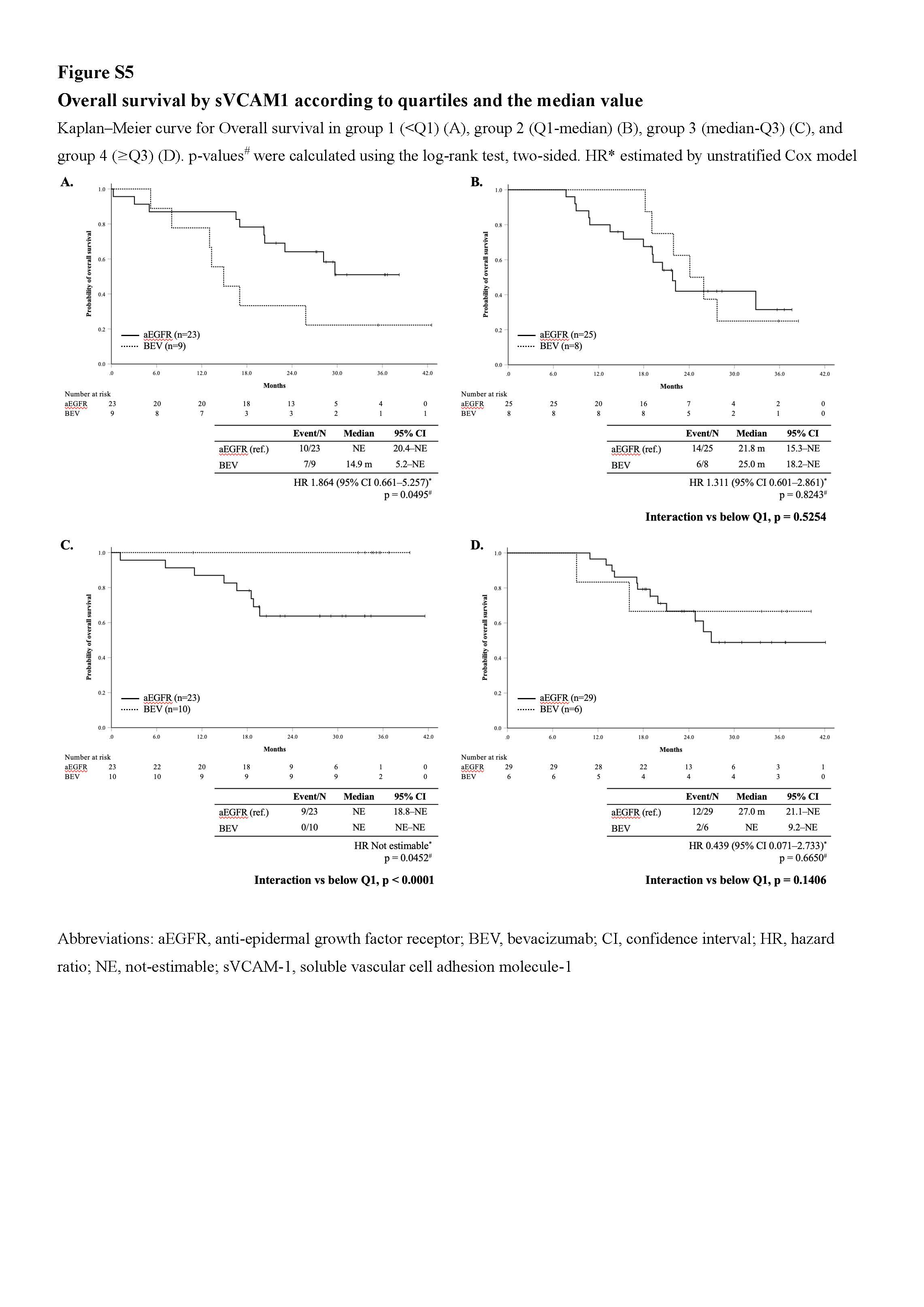

Supplement: Supplementary file 5 — Figure S5: [file CAM4-12-18702-s002.jpg]
