# Supplementary material for: Role of plasma angiogenesis factors in the efficacy of first‐line chemotherapy combined with biologics in RAS wild‐type metastatic colorectal cancer: Results from the GI‐SCREEN CRC‐Ukit study
Source: Cancer Med. 2023 Aug 28;12(18):18702–16. doi: 10.1002/cam4.6486 (PMC10557901; doi:10.1002/cam4.6486)
Supplement: Supplementary file 6 — Table S1. [file CAM4-12-18702-s005.docx]

**Table S1. Response rate in the efficacy analysis set**

|  | N | CR | PR | SD | PD | NE | RR (%) | (95% CI) |
| --- | --- | --- | --- | --- | --- | --- | --- | --- |
| aEGFR | 97 | 3 | 69 | 17 | 5 | 3 | 74.2 | 65.5–82.9 |
| BEV | 31 | 0 | 15 | 15 | 1 | 0 | 48.4 | 30.8–66.0 |

Odds ratio 0.326 (95% CI 0.141–0.753), p = 0.014

Abbreviations: aEGFR, anti-epidermal growth factor receptor; BEV, bevacizumab; CR, complete response; PR, partial response; SD, stable disease; PD, progressive disease; NE, not evaluable; RR, response rate; CI, confidence interval

**Table S2. Response rate by each baseline plasma angiogenesis factor in the efficacy analysis set**

|  |  | Below median | | | | | Above median | | | | | Interaction p |
| --- | --- | --- | --- | --- | --- | --- | --- | --- | --- | --- | --- | --- |
|  |  | N | RR (%) | 95% CI | Odds rate  (95% CI) | p-value | N | RR (%) | 95% CI | Odds rate  (95% CI) | p-value |  |
| Angiopoietin-2 | aEGFR  BEV | 47  15 | 76.6  53.3 | 64.5–88.7  28.1–78.6 | 0.349 (0.103–1.181) | 0.108 | 50  16 | 72.0  43.8 | 59.6–84.4  19.4–68.1 | 0.302 (0.094–0.969) | 0.068 | 0.120 |
| HGF | aEGFR  BEV | 48  15 | 68.8  53.3 | 55.6–81.9  28.1–78.6 | 0.519 (0.159–1.697) | 0.355 | 49  16 | 79.6  43.8 | 68.3–90.9  19.4–68.1 | 0.199 (0.060–0.667) | 0.011 | 0.576 |
| **IL-8** | aEGFR  BEV | 43  19 | 62.8  42.1 | 48.3–77.2  19.9–64.3 | 0.431 (0.143–1.296) | 0.169 | 54  12 | 83.3  58.3 | 73.4–93.3  30.4–86.2 | 0.280 (0.072–1.083) | 0.111 | **0.086** |
| **PlGF** | aEGFR  BEV | 51  12 | 78.4  58.3 | 67.1–89.7  30.4–86.2 | 0.385 (0.102–1.452) | 0.162 | 46  19 | 69.6  42.1 | 56.3–82.9  19.9–64.3 | 0.318 (0.105–0.962) | 0.052 | **0.019** |
| VEGF-A | aEGFR  BEV | 45  17 | 75.6  47.1 | 63.0–88.1  23.3–70.8 | 0.288 (0.089–0.927) | 0.065 | 52  14 | 73.1  50.0 | 61.0–85.1  23.9–76.2 | 0.368 (0.109–1.240) | 0.117 | 0.505 |
| VEGF-D | aEGFR  BEV | 44  19 | 79.5  52.6 | 67.6–91.5  30.2–75.1 | 0.286 (0.089–0.912) | 0.038 | 53  12 | 69.8  41.7 | 57.5–82.2  13.8–69.6 | 0.309 (0.085–1.121) | 0.095 | 0.125 |
| OPN | aEGFR  BEV | 42  20 | 76.2  50.0 | 63.3–89.1  28.1–71.2 | 0.313 (0.101–0.966) | 0.048 | 55  11 | 72.7  45.5 | 61.0–84.5  16.0–74.9 | 0.313 (0.083–1.178) | 0.090 | 0.491 |
| **sNeuropillin-1** | aEGFR  BEV | 48  15 | 79.2  53.3 | 67.7–90.7  28.1–78.6 | 0.301 (0.088–1.029) | 0.092 | 49  16 | 69.4  43.8 | 56.5–82.3  19.4–68.1 | 0.343 (0.108–1.094) | 0.080 | **0.057** |
| **sVEGFR-1** | aEGFR  BEV | 47  18 | 83.0  55.6 | 72.2–93.7  32.6–78.5 | 0.256 (0.077–0.852) | 0.050 | 50  13 | 66.0  38.5 | 52.9–79.1  12.0–64.9 | 0.322 (0.091–1.137) | 0.111 | **0.016** |
| **sVEGFR-2** | aEGFR  BEV | 48  15 | 81.3  53.3 | 70.2–92.3  28.1–78.6 | 0.264 (0.076–0.918) | 0.043 | 49  16 | 67.3  43.8 | 54.2–80.5  19.4–68.1 | 0.377 (0.119–1.196) | 0.139 | **0.031** |
| **sVEGFR-3** | aEGFR  BEV | 46  17 | 78.3  58.8 | 66.3–90.2  35.4–82.2 | 0.397 (0.120–1.309) | 0.199 | 51  14 | 70.6  35.7 | 58.1–83.1  10.6–60.1 | 0.231 (0.066–0.806) | 0.027 | **0.034** |
| TSP-2 | aEGFR  BEV | 46  17 | 76.1  52.9 | 63.8–88.4  29.2–76.7 | 0.354 (0.110–1.138) | 0.120 | 51  14 | 72.5  42.9 | 60.3–84.8  16.9–68.8 | 0.284 (0.083–0.965) | 0.056 | 0.190 |
| sICAM-1 | aEGFR  BEV | 44  18 | 70.5  50.0 | 57.0–83.9  26.9–73.1 | 0.419 (0.136–1.296) | 0.152 | 53  13 | 77.4  46.2 | 66.1–88.6  19.1–73.3 | 0.251 (0.071–0.890) | 0.040 | 0.843 |
| sVCAM-1 | aEGFR  BEV | 48  17 | 66.7  41.2 | 53.3–80.0  17.8–64.6 | 0.350 (0.112–1.091) | 0.087 | 49  14 | 81.6  57.1 | 70.8–92.5  31.2–83.1 | 0.300 (0.083–1.081) | 0.078 | 0.307 |
| TIMP-1 | aEGFR  BEV | 43  19 | 72.1  47.4 | 58.7–85.5  24.9–69.8 | 0.348 (0.114–1.068) | 0.085 | 54  12 | 75.9  50.0 | 64.5–87.3  21.7–78.3 | 0.317 (0.087–1.154) | 0.089 | 0.996 |

For interferon-γ and IL-6, more than half of the patients were below the lower detection limit (2.00 pg/mL), and no analysis was performed.

Abbreviations: aEGFR, anti-epidermal growth factor receptor; BEV, bevacizumab; RR, response rate; CI, confidence interval; HGF, hepatocyte growth factor; IL, interleukin; PlGF, placental growth factor; VEGF, vascular endothelial growth factor; OPN, osteopontin; sVEGFR, soluble vascular endothelial growth factor-receptor; TSP-2, thrombospondin-2; sICAM-1, soluble intercellular adhesion molecule-1; sVCAM-1, soluble vascular cell adhesion molecule-1; TIMP-1, tissue inhibitor of metalloproteinase-1

**Table S3. Baseline plasma angiogenesis-related factors in the efficacy analysis set**

| **(pg/mL)** |  | **All (n=133)** | **aEGFR (n=100)** | **BEV (n=33)** | ***p*-value** |
| --- | --- | --- | --- | --- | --- |
| Angiopoietin-2 | Median  (range) | 1450  (346–10200) | 1465  (346–10200) | 1450  (505–6540) | 0.356 |
| HGF | Median  (range) | 114  (< 60.0–1110) | 115  (< 60.0–1110) | 109  (< 60.0–437) | 0.967 |
| IFN-γ | Median  (range) | < 2.00  (< 2.00–61.4) | < 2.00  (< 2.00–64.2) | < 2.00  (< 2.00–52.3) | 0.131 |
| **IL-6** | Median  (range) | < 2.00  (< 2.00–61.4) | < 2.00  (< 2.00–61.4) | < 2.00  (< 2.00–28.6) | **0.025** |
| **IL-8** | Median  (range) | 8.03  (< 2.00–239) | 10.8  (< 2.00–147) | 3.61  (< 2.00–239) | **0.054** |
| PlGF | Median  (range) | 6.39  (< 3.00–588) | 5.94  (< 3.00–588) | 7.94  (< 3.00–34.6) | 0.434 |
| VEGF-A | Median  (range) | 116  (< 30.0–1500) | 124  (< 30.0–1500) | 97.1  (< 30.0–662) | 0.128 |
| VEGF-D | Median  (range) | 263  (< 60.0–814) | 264  (< 60.0–696) | 224  (< 60.0–814) | 0.245 |
| **OPN** | Median  (range) | 12100  (996–73100) | 12750  (996–73100) | 9210  (2730–39300) | **0.024** |
| sNeuropillin-1 | Median  (range) | 294000  (82800–1000000) | 294000  (82800–1000000) | 287000  (119000–588000) | 0.950 |
| sVEGFR1 | Median  (range) | 1350  (52.7–7810) | 1420  (95.9–7810) | 1240  (52.7–3600) | 0.159 |
| sVEGFR2 | Median  (range) | 11300  (2450–26700) | 11200  (2450–26700) | 11600  (5000–16300) | 0.983 |
| sVEGFR3 | Median  (range) | 22500  (1500–118000) | 22900  (1500–118000) | 21000  (1500–48100) | 0.315 |
| TSP-2 | Median  (range) | 20000  (500–235000) | 20950  (500–235000) | 19600  (500–187000) | 0.407 |
| sICAM-1 | Median  (range) | 403000  (76500–8000000) | 412000  (93110–8000000) | 366000  (76500–2320000) | 0.100 |
| sVCAM-1 | Median  (range) | 1020000  (494000–5770000) | 10250000  (494000–5770000) | 1010000  (517000–1730000) | 0.527 |
| **TIMP-1** | Median  (range) | 307000  (130000–1850000) | 318500  (130000–1850000) | 243000  (132000–1580000) | **0.032** |

Abbreviations: aEGFR, anti-epidermal growth factor receptor; BEV, bevacizumab; HGF, hepatocyte growth factor; IFN-γ, interferon-γ; IL, interleukin; PlGF, placental growth factor; VEGF, vascular endothelial growth factor; OPN, osteopontin; sVEGFR, soluble vascular endothelial growth factor-receptor; TSP-2, thrombospondin-2; sICAM-1, soluble intercellular adhesion molecule-1; sVCAM-1, soluble vascular cell adhesion molecule-1; TIMP-1, tissue inhibitor of metalloproteinase-1
